# Prebiotic food intake and biochemical measures in diabetic patients: A cross-sectional study from the Sabzevar Persian Cohort

**DOI:** 10.1371/journal.pone.0332029

**Published:** 2025-10-01

**Authors:** Rahil Mahmoudi, Maral Nabaee, Akram Kooshki, Saba Shourideh Yazdi, Mahboubeh Neamatshahi, Elaheh Foroumandi

**Affiliations:** 1 Iranian Research Center on healthy Aging, Sabzevar University of Medical Sciences, Sabzevar, Iran; 2 Student Research Committee, Sabzevar University of Medical Sciences, Sabzevar, Iran; 3 Non-Communicable Diseases Research Center, Sabzevar University of Medical Sciences, Sabzevar, Iran; University of Jeddah, SAUDI ARABIA

## Abstract

**Background:**

With the high prevalence of diabetes worldwide and the known benefits of functional foods in controlling diabetes, this study aimed to explore the connection between prebiotic food intake and biochemical indices in diabetic patients.

**Methods:**

This cross-sectional study was conducted on 504 participants with type 2 diabetes who were part of the Sabzevar Persian Cohort study that was initiated in February 2018. A 148-items food frequency questionnaire was utilized to assess the daily amount of prebiotic food consumption. Blood serum samples were collected from participants to measure fasting blood sugar levels, lipid profile, and kidney function indicators. Additionally, anthropometric measurements were taken following standard protocols. Statistical analyses were performed using SPSS version 20, with correlation tests adjusted for confounders, and significance set at p < 0.05.

**Results:**

This study involved 44.9% male and 55.1% female participants, with an average age of 54.81 ± 7.65 years. A significant correlation was observed between soybean consumption and serum low density lipoprotein (LDL) status (R = −0.110, P = 0.014). Moreover, a higher intake of banana was linked to lower blood urea nitrogen (BUN) levels (R = −0.109, P = 0.015). Furthermore, the consumption of honey exhibited a negative association with both systolic blood pressure (BP) (R = −0.106, P = 0.018) and diastolic BP (R = −0.132, P = 0.003). Green peas intake was also inversely associated with DBP (R = −0.092, P = 0.039).

**Conclusion:**

This study found a positive correlation between the regular intake of prebiotic foods and improved management of BUN, LDL, and BP in individuals with type 2 diabetes. However, further mechanistic studies are necessary to better understand the potential causal effects of prebiotic foods on metabolic health in this population.

## Introduction

Type 2 diabetes is a metabolic disorder characterized by elevated blood glucose levels, insulin resistance, or relative insulin deficiency [1]. Worldwide, diabetes is one of the main leading causes of morbidity and mortality. Globally, around 7.7% of adults between the ages of 25 and 64 have been diagnosed with type 2 diabetes, with projections estimating that this number could rise to 592 million by the year 2035 [2]. In Iran, 40% of deaths occurred due to type 2 diabetes [3], and it is estimated that approximately 9.2 million of Iranian population suffered from diabetes by 2030 [4]. Diabetes along with the complications caused by the disease, increase the economic burden of societies to approximately 1.8% of gross domestic product (GDP) [5]. In USA, the health care expenditure on diabetes was reported 412.9 dollars in 2022 [6].

Diabetic patients experience elevated blood glucose levels in their bodies, leading to the spontaneous oxidation of body sugars. This process results in the production and accumulation of reactive oxygen species (ROS) and advanced glycosylation end products (AGEs) within their bodies [7,8]. These oxidative and inflammatory conditions contribute to insulin resistance, which is a key factor in the development of complications associated with type 2 diabetes [9]. Furthermore, the disrupted metabolic patterns and heightened levels of inflammatory markers in diabetic patients play crucial roles in the pathogenesis of macro vascular complications [10].

The pathogenesis of diabetes is complex and various genetic and environmental factors are involved. A healthy lifestyle involving adequate physical activity and healthy diet are contributed to low risk of diabetes occurrence [11]. A diet rich in whole grains that consists a high amounts of fiber, and low saturated fat is associated with reduced risk of diabetes by 18% [12]. Further, regular legume consumption may play an effective role in reducing the levels of fasting blood glucose (FBG), glycated hemoglobin (HbA1c), and 2-hour post prandial glucose in diabetic patients [13]. Mostly a healthy diet is rich sources of foods with low glycemic index (GI) including whole grains, legumes, and vegetables that are high in various prebiotics contents [14].

Prebiotics are indigestible substances that aren’t broken down in the upper digestive tract but are fermented by bacteria in the large intestine. The most important prebiotics are fructo-oligosaccharides (FOS), Insulin, and galacto-oligosaccharides (GOS). Sources of prebiotics include seeds, whole grains, legumes, chicory root, artichokes, onions, garlic, and certain vegetables [15]. These foods can promote the growth of beneficial bacteria like bifidobacteria and lactobacilli in the gut, leading to positive changes in gut microbiota metabolism and function. Additionally, increasing dietary fiber intake through prebiotics may trigger satiety hormones, aiding in appetite control and potential weight management [16,17]. Furthermore, a healthy balance of gut microbiota is related to low risk of obesity and metabolic related disorders [18].

Several studies have demonstrated that FOS intake can reduce FBG, cholesterol, and blood pressure levels [19,20]. For example, Bock et al. reported that probiotic and prebiotic supplementation in diabetic patients reduced FBG, total cholesterol, and triglycerides, though no significant effect was observed on HbA1c [21]. While substantial evidence supports the effects of probiotics and synbiotics on biochemical indices, limited studies have specifically investigated the association between the regular consumption of natural prebiotic foods and multiple metabolic parameters [22–24].

Importantly, previous studies have primarily focused on glycemic control, inflammatory markers, or gut microbiota changes, often with limited sample sizes or study durations. In contrast, the present study aims to fill this research gap by evaluating the correlation between the intake of various prebiotic foods including soybeans, banana, green peas, and honey—and a broader range of biochemical indicators such as blood urea nitrogen (BUN), low-density lipoprotein (LDL), and blood pressure in a large cohort of individuals with type 2 diabetes. Given the high prevalence of type 2 diabetes and variations in gut microbiota across populations, this research offers novel insights into the role of dietary prebiotics in managing kidney function and cardiovascular risk factors in diabetic patients.

## Methods and materials

This cross-sectional study was conducted on the individuals referred to the Sabzevar Persian Cohort Center. Totally, 4242 subjects over the age 35 years were participated in the Sabzevar Persian Cohort study that was initiated in February 2018. Among them, 590 subjects were diagnosed with diabetes, . The inclusion criteria included adult patients with the age range of 35–70 years and a history of diabetes for at least two years (in order to definitively diagnose the disease by specialist), while the exclusion criteria encompassed patients with gastrointestinal or kidney diseases and individuals who were using probiotics, prebiotics, or synbiotics supplements. All participants were asked to sign informed consent forms. It was explained to all the subjects that the information obtained from them will remain confidential and will be used only for conducting the study.

At baseline, the 148-item food frequency questionnaire (FFQ) was utilized to assess daily prebiotic food consumption, including items such as garlic, onion, honey, apple, banana, tomato, wheat, soybean, lentils, beans, and green peas [25]. The quantities of these prebiotic foods were recorded in grams per day. Additionally, 5 cc of blood serum were collected for enzymatic analysis of fasting blood sugar (FBS), triglycerides (TG), total cholesterol (TC), and high-density lipoprotein-cholesterol (HDLc) levels. Commercial kits from Pars Azemoon Co., Tehran, Iran, and a Hitachi 717 auto-analyzer from Boehringer Mannheim Diagnostics, Mannheim, Germany, were employed for this analysis. Serum low-density lipoprotein cholesterol (LDL-c) levels were estimated using the Friedewald equation [26].

Participants’ body weight was measured with minimal clothing and without shoes, using a SECA digital scale with an accuracy of 100 grams. Height was measured using a non-expandable tape measure mounted on the wall with an accuracy of 0.1 cm, in a standing position without shoes, while the shoulders were in a normal condition. The Body Mass Index (BMI) was calculated using the formula of weight (in kilograms) divided by the square of height (in meters). The protocol of this study has been approved by the ethics committee of Sabzevar University of Medical Sciences (IR.MEDSAB.REC.1401.83).

Statistical analysis was performed using SPSS version 20. The normality of data was assessed using the Shapiro–Wilk test. Depending on data distribution, Pearson or Spearman correlation tests were applied to evaluate relationships between prebiotic food intake and biochemical markers. Missing data were minimal; participants with incomplete data for key variables were excluded from respective analyses to ensure data integrity. All analyses were adjusted for potential confounders including age, gender, age at diabetes diagnosis, BMI, and history of hypertension. Results were presented as mean ± standard deviation (SD), with correlation coefficients, and p-values. A p-value < 0.05 was considered statistically significant.

## Results

### Study subjects

From 590 subjects who were diagnosed with diabetes, 504 patients were eligible to participate in the study. Totally, 56 people had a history of less than 2 years of disease, 15 people had kidney disease, and the rest had provided incomplete data.

In this cross-sectional study, 43.8% (221 individuals) of the participants were male, while 56.2% (283 individuals) were female. The demographic and anthropometric characteristics of the study participants can be found in Table 1. The mean age was 54.81 ± 7.65 years with a BMI of 29.37 ± 4.74 kg/m^2^.

**Table 1:** Demographic and anthropometric characteristics of study participants (N = 504).

Variables		Mean	SD
Age (years)		54.81	7.65
Gender^*^	Male	221	43.8
	Female	283	56.2
Age of diabetes diagnosis (years)		47.06	9.65
History of high blood pressure^*^	Yes	245	48.6
	No	259	51.4
Height (cm)		162.07	8.98
Body weight (Kg)		75.22	13.81
Waist circumference (cm)		96.80	10.23
Hip circumference (cm)		105.39	8.25
Body mass index (kg/m^2^)		29.37	4.74

* Expressed by number (percent).

### Prebiotic foods intake

As indicated in Table 2, tomato was the most consumed prebiotic food, with subjects consuming an average of 168.55 ± 5.15 grams per day. In contrast, garlic had the lowest consumption levels as a prebiotic food, with an average intake of 1.44 ± 0.10 grams per day (Table 2).

**Table 2:** Daily consumption of prebiotic foods by participants (grams) (n = 504).

Variables	Mean	SE
Garlic	1.44	0.10
Onion	82.15	2.26
Honey	1.86	0.16
Apple	106.53	4.94
Banana	6.03	0.41
Tomato	168.55	5.15
Wheat	12.50	0.92
Soybean	2.35	0.19
Lentils	12.12	0.73
Beans	7.41	0.37
Green peas	4.54	0.33

### Biochemical characteristics

In Table 3, the biochemical levels of the study subjects are presented. It is notable that the FBS level was outside the normal range, with a measurement of 173.37 ± 70.51 mg/dL. However, the levels of other biochemical markers were within normal ranges.

**Table 3:** Serum levels of biochemical variables and blood pressure of study subjects (n = 504).

Variables	Mean	SD
FBS (mg/dL)	173.37	70.52
TG (mg/dL)	173.11	111.74
Chol (mg/dL)	185.56	45.78
HDL (mg/dL)	51.81	10.51
LDL (mg/dL)	101.06	36.45
Cr (mg/dL)	1.12	0.24
BUN (mg/dL)	14.43	4.13
GFR (ml/min/1.73m^2^)	82.26	15.96
SBP (mmHg)	112.75	15.42
DBP (mmHg)	72.11	9.4

### Association of prebiotic foods with biochemical indices

**Fig 1:**
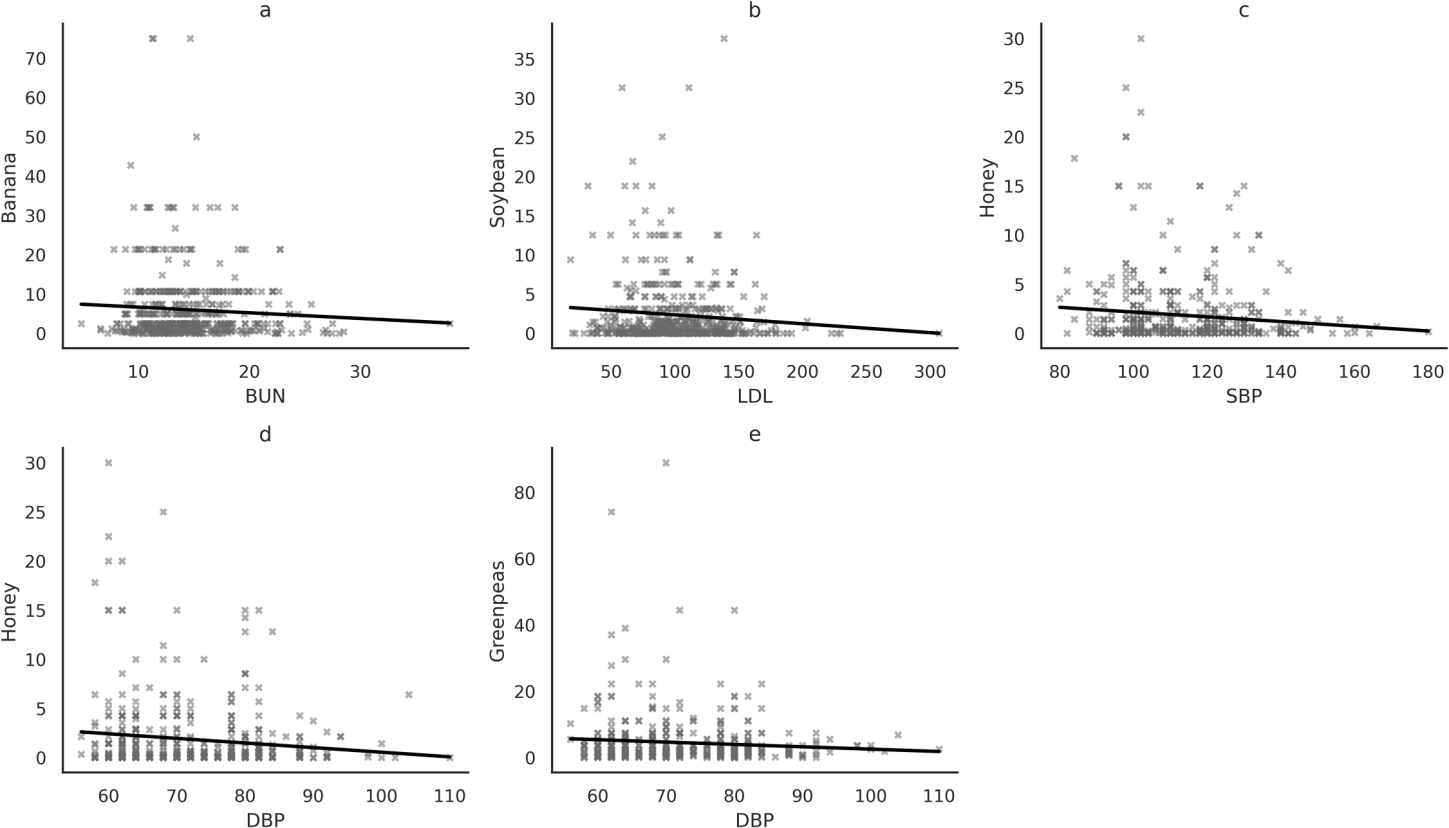
The correlation of prebiotic foods with biochemical indices, (a): Banana and BUN (R = −0.109, P = 0.015). (b): Soybean and LDL (R = −0.110, P = 0.014). (c): Honey and SBP (R = −0.106, P = 0.018). (d): Honey and DBP (R = −0.132, P = 0.003). (e): Greanpeas and DBP (R = −0.092, P = 0.039). All the analysis was adjusted for BMI, age, gender, has HTN, and DM start age as the confounders.

Analysis in Figure 1 revealed significant associations between banana and BUN levels (R = −0.109, P = 0.015) (Fig 1(a)). Additionally, higher intake of soybean was linked to lower LDL status (R = −0.110, P = 0.014) (Fig 1(b)). Notably, honey consumption showed inverse correlations with both SBP (R = −0.106, P = 0.018) and DBP (R = −0.132, P = 0.003) (Fig 1(c and d)). Furthermore, there was an inverse association between greanpeas intake and DBP level (R = −0.092, P = 0.039) (Fig 1(e)).

## Discussion

The findings of this cross-sectional study involving diabetic patients from the Sabzevar Persian Cohort study revealed a significant negative association between soybean intake and serum LDL. Additionally, a negative correlation was observed between banana consumption and BUN levels. Moreover, the consumption of honey was found to be inversely associated with SBP and DBP. The negative association was also detected between green peas intake and DBP levels.

The study has shown that a high intake of soybeans was associated with lower serum LDL levels.

Previous studies have demonstrated the potential benefits of soy consumption on lipid profile of various populations. A meta-analysis of 46 randomized control trials has reported a reduction in TG, TC, and Apo-B, and LDL levels in postmenopausal women by consumption of soy [27]. A same report was also shown in Baranska et al., study that was done on 2305 postmenopausal women [28]. A meta-analysis on diabetic patients has shown that both soy protein and extracted isoflavone intake can effectively reduce lipid profile, but not FBG [28]. Yang et al., has also not detected any association between soy intake and serum glucose levels [29]. Inversely, a meta-analysis on 8 trials revealed significant changes in FPG, fasting serum insulin, and HbA1C, as well as TG, TC, LDL, and HDL by soy supplementation [30]. Other research studies have reported positive effects of soy consumption on inflammatory factors [31,32]. The inconsistencies in the results could be attributed to differences in the levels and duration of soy intake, as well as the baseline serum lipid indices concentrations of the subjects. Various mechanisms may contribute to the effects of soybean intake on lipid profile. These include the impact of soy consumption on lipid metabolism, blood pressure reduction, activation of ACE-inhibitory pathways, inflammation regulation, and tyrosine kinase inhibition, among others [15,^33^-^34^–]. Moreover, the structural similarity between soy isoflavones and endogenous 17-β-estradiol suggests that isoflavones, by binding to estrogen receptors, lead to gene activation and beneficial effects on glucose and lipid metabolism [35]. Overall, it can be inferred that maintaining a diet high in soybean consumption could be beneficial in reducing LDL prior to the development of cardiovascular disorders in diabetic patients.

Honey intake was related to lower SBP and DBP in current study. In line with our study, intake of 20 ml honey by 100 participants was found to be linked to lower SBP and DBP [36]. Another research paper on 50 healthy female subjects has reported that consumption of 20 ml honey has a negative association with SBP [37], although the exact mechanism behind this remains unknown. However, contrary to our findings, in a study on 60 Iranian healthy male subjects there was no significant association between daily intake of 70 gram honey for 6 week and SBP or DBP. The limited sample size of this study may have influenced this outcome [38]. Further, a systematic review study has investigated that honey intake has not any beneficial effects on metabolic indices of healthy subjects, as well as high intake of honey may increase serum glucose status among diabetic patients [38]. The results of this study may be due to high heterogeneity, so it was not possible to perform meta-analysis on the results. Taken altogether, the differences in the mentioned results may be related to the type of consumed honey and also the amount of fructose and the ratio of fructose to glucose in honey. The existence of food adulteration and the addition of fructose to honey cause intervention in the natural nature of this food and it therapeutic properties. The relationship between fructose intake and increased serum triglycerides has been proven in past studies. Also, increasing the amount of fructose in honey increases its glycemic index, which itself causes the lack of control of blood sugar and metabolic factors in people with diabetes [39,40]. More extensive studies are needed to determine the exact mechanism of blood pressure control thorough honey intake among diabetic patients. Honey is a natural product that is rich in trace elements, such as various antioxidants, zinc, copper, and other unidentified substances. The presence of nonabsorbable and alcoholic sugars in honey results in the production of high prebiotic compounds. Furthermore, the antioxidant content of honey, including quercetin, caffeic acid phenethyl ester, kaempferol, acacetin, and galangin, may play a role in improving blood pressure [41]. The cardio-protective benefits associated with honey may be attributed to its significant role in reducing markers of cardiac damage, such as creatine kinase-MB (CK-MB), lactate dehydrogenase (LDH), aspartate aminotransferase (AST), and alanine aminotransferase (ALT) [42,43].

Banana consumption was related to lower BUN levels in this study. There is not enough literature to evaluate the effects of banana intake on renal function of diabetic patients. In an animal study on 30 male rats has shown that consumption of dried banana male bud is contributed to elevation of kidney function including uric acid and urea nitrogen [44]. In rats with type 1 diabetes, green banana intake has beneficial effects on liver function and lipid profile. This study has not reported any variation in serum creatinine status after consumption of banana by rats [45]. A human study has reported that daily intake of 250 and 500 grams of banana for 4 weeks among diabetic patients was associated with plasma adiponectin, but not lipid profile, kidney and liver function parametes, or blood glucose [46]. Banana, as a functional food is a main source of flavonoids and indigestible fractions that are contributed to beneficial effects on gut regulation and appetite control [47]. Mechanisms remain to be elucidated.

The negative association of green peas intake and DBP levels was shown in this study. In line with our study, another study that was done on 4680 US adults with an age range of 40–59 years has reported an inverse association between green peas intake and blood pressure [48]. Both the soluble and insoluble fiber contents in green peas may contributes to improving endothelial vasodilation, angiotensin balance, and consequently reduced BP levels [49]. Future investigations should delve deeper into the effects of green peas intake on DBP, taking into account factors such as the subjects’ dietary pattern, initial energy intake levels, and the composition of their basic intestinal microflora.

Although, the large sample size of this study is a main strength, there is some limitation that should be noted. Considering that a food frequency questionnaire was used to determine the intake of prebiotic foods, underreporting and over-reporting of participants are common, especially in people with abnormal BMI. Further, measurement of serum insulin and HbA1c levels could be helpful in obtaining more reliable results, which was not possible for us due to the budget constraints.

## Conclusion

In conclusion, this study found a positive association between the consumption of prebiotic foods such as soybeans, banana, green peas, and honey and improvements in lipid profile, kidney function, and blood pressure in diabetic patients. However, these findings do not establish causality. Further research is warranted to determine the optimal daily intake of prebiotic foods and to explore the underlying mechanisms responsible for these effects.

## Supporting information

S1 Data Dataset. (SAV)
